# Knowledge–practice gap in leishmaniasis among medical students in an endemic region: a multi-university cross-sectional study

**DOI:** 10.3389/fpubh.2026.1864876

**Published:** 2026-07-06

**Authors:** Mahmoud Yousef, Hamzeh Yacoub, Malak Badawi, Anhar Alhawarin, Sara Ghaith, Miar Haj-Abdalrahman, Israa Namarneh, Mohammad Abueisa, Manar Shukri Jaber, Obida Hmedat, Asad Omarion

**Affiliations:** 1 Faculty of Medicine, Al-Quds University, Jerusalem, Palestine; 2 Faculty of Medicine, Arab American University of Palestine, Ramallah, Palestine; 3 Faculty of Medicine, Palestine Polytechnic University, Hebron, Palestine; 4 Faculty of Medicine, Mansoura University, Mansoura, Egypt; 5 Faculty of Medicine, Alexandria University, Alexandria, Egypt

**Keywords:** endemic diseases, knowledge–attitude–practice, leishmaniasis, medical students, neglected tropical diseases, public health

## Abstract

Leishmaniasis remains a major neglected tropical disease in endemic regions, where early recognition, reporting, and management are essential for effective control. Medical students represent a critical link between clinical practice and public health surveillance. However, gaps in knowledge, attitudes, and practices (KAP), particularly the translation of knowledge into actionable behavior, remain underexplored. A multi-university cross-sectional study was conducted among medical students in Palestine using a structured, self-administered online questionnaire. Data were collected between January 2026 and February 2026. The survey assessed knowledge, attitudes, and practices related to leishmaniasis, along with relevant demographic and educational factors. Knowledge, attitude, and practice composite scores were analyzed as both continuous and categorical variables using predefined thresholds: poor (<60%), moderate (60%−79%), and good (≥80%) of the maximum possible score. Adjusted regression models were used to examine the relationship between knowledge and attitudes and to assess the joint influence of knowledge and attitudes on practices. In contrast, only 12.0% of participants were classified as having good attitude/professional responsibility scores, and only 9.1% had good practice scores, while 51.5% were classified as having poor practice scores. In adjusted models, knowledge was not independently associated with attitude score (B per 10-percentage-point increase = 0.04; 95% CI: −0.03 to 0.11; *p* = 0.227). When knowledge and attitude were entered jointly, neither knowledge nor attitude independently predicted practice score. A total of 408 participants were included in the analysis. The mean knowledge score was 72.98% (95% CI: 70.60–75.37), with 44.4% classified as having “good” knowledge. Despite relatively high knowledge levels, reporting practices were markedly inadequate, with only 7.6% consistently reporting suspected cases and 24.5% unaware of reporting systems. Knowledge was significantly higher among clinical-stage students and those who had completed parasitology or infectious diseases coursework, the latter demonstrating the strongest independent association with higher knowledge (β = +19.55 percentage points, *p* < 0.001). Endemic-area exposure and prior awareness of leishmaniasis were also significant predictors. Notably, knowledge showed weak correlation with both attitudes and practices, indicating a disconnect between theoretical understanding and behavioral application. While medical students demonstrated generally adequate knowledge of leishmaniasis, a substantial gap exists between knowledge and practical application, particularly in public health reporting. This disconnect represents a critical vulnerability in the clinical–public health interface. Addressing this gap requires targeted educational strategies that integrate clinical training with surveillance awareness and competency-based learning to improve both patient outcomes and disease control efforts in endemic settings.

## Introduction

Leishmaniasis is a parasitic disease caused by various species of protozoan parasites belonging to the genus *Leishmania*. Transmission occurs through the bite of infected phlebotomine sandflies, which introduce the parasite into the host during blood feeding (1). The disease manifests in three main forms: cutaneous leishmaniasis (CL), mucocutaneous leishmaniasis, and visceral leishmaniasis (VL) (2). The most common form is CL, characterized by ulcerative skin lesions that usually occur on exposed areas of the body and may lead to disfigurement (3). These lesions can result in significant morbidity, including disability, social stigma, and permanent scarring (3).

Globally, an estimated 700,000 to 1 million new cases are reported every year (4). The disease disproportionately affects populations in low-income settings, where living conditions are often difficult. Risk factors such as malnutrition, population displacement, inadequate housing, immunosuppression, and limited financial resources contribute to increased vulnerability (4).

Leishmaniasis is endemic in several regions of the Middle East, with many countries reporting ongoing transmission and periodic outbreaks (5). In Palestine, leishmaniasis constitutes the most prevalent endemic parasitic disease among NTDs. According to the Palestinian Ministry of Health surveillance data and published spatiotemporal analyses, hundreds of cutaneous leishmaniasis cases are reported annually in the West Bank, with Jericho, Jenin, and Tubas emerging as persistently high-burden districts (6). Projections based on climate change models suggest that the geographic range of transmission may expand significantly through 2060, further amplifying the public health significance of the disease (6). Despite ongoing surveillance and control efforts, leishmaniasis continues to pose significant public health challenges in the region (6).

Despite the endemicity of leishmaniasis in Palestine and the availability of epidemiological data, limited attention has been directed toward assessing the level of understanding of the disease among Palestinian medical students (6). A systematic search of PubMed, Scopus, and Google Scholar using the terms “leishmaniasis,” “knowledge,” “Palestine,” and “medical students” identified no prior KAP studies conducted specifically among Palestinian medical students, establishing a clear research gap. Since medical students widely represent the interface between clinical medicine and public health surveillance, knowledge, attitude, and practices (KAP) gaps among future physicians may directly impair early detection, reporting, and control of endemic neglected tropical diseases (NTDs) globally (7). Existing research has largely focused on disease epidemiology, transmission dynamics, and risk factors, with less emphasis on the preparedness of future healthcare providers (10, 11). Additionally, variations in medical curricula and levels of clinical exposure across universities may contribute to inconsistencies in students' knowledge and perceptions of leishmaniasis (12, 13). Future physicians play a critical role not only in clinical diagnosis and management but also in disease surveillance, reporting, and public health response (7, 10, 11). Inadequate preparedness at the undergraduate level may contribute to delayed diagnosis, underreporting, and the continuation of transmission cycles. Addressing these gaps is essential for strengthening medical education regarding the disease and improving disease prevention and control strategies. Therefore, further research is needed to evaluate the KAP of medical students and to identify areas requiring targeted educational interventions. Thus, evaluating KAP among medical students in endemic settings provides insight into systemic gaps in medical education that may be relevant to other regions facing similar epidemiological challenges.

Importantly, this study does not merely assess knowledge levels but seeks to operationalize the knowledge–practice gap, defined here as the measurable discordance between a student's assessed knowledge score and their self-reported clinical or public health behaviors (specifically: case reporting frequency, PPE use, and appropriate diagnostic action). In the context of leishmaniasis, this gap is of particular public health importance because underreporting of cases, a behavioral failure, directly impairs disease surveillance and outbreak response, even when clinical knowledge is adequate (7). The knowledge–practice gap framework applied here draws on established behavioral theories, including the Knowledge–Behavior Gap model (16) and the Health Belief Model (17), which posit that knowledge is a necessary but insufficient condition for behavior change without perceived responsibility, self-efficacy, and structural enablers. This theoretical framing differentiates the present study from descriptive KAP surveys and provides a basis for translating findings into targeted educational interventions.

Previous KAP studies on leishmaniasis among medical students have primarily been conducted in countries such as Iran and Morocco, demonstrating variable knowledge levels and persistent gaps in practice (10–12, 14, 15). However, comparable data from Palestine and similar endemic settings remain limited, highlighting the need for context-specific evaluation.

This study aims to assess the knowledge, attitudes, and practices regarding leishmaniasis among medical students in Palestine through a multi-university survey, while also contextualizing the findings within the regional epidemiological landscape.

## Methodology

### Study design and setting

A cross-sectional survey was conducted among undergraduate medical students enrolled in eight Palestinian universities spanning the West Bank, Gaza Strip, and East Jerusalem, as well as a subset studying at Egyptian institutions, representing a total of 10 universities across the region, to assess knowledge, attitudes, and practices (KAP) regarding leishmaniasis and related professional responsibilities. The study setting comprised eight Palestinian universities with medical faculties (Al-Quds University, Palestine Polytechnic University, An-Najah National University, Arab American University of Palestine, Hebron University, Islamic University of Gaza, and others), spanning the West Bank, East Jerusalem, and the Gaza Strip, as well as a subset enrolled at Egyptian institutions (Mansoura University and Alexandria University). Palestinian medical education follows a 6-year program structure, with the first 3 years dedicated to pre-clinical sciences (including parasitology and infectious diseases coursework) and the final 3 years to clinical rotations in affiliated hospitals. The precise total enrollment was not available from public institutional records at the time of the study. Data collection was conducted between January 2026 and February 2026. The study used a structured, self-administered questionnaire delivered primarily through an online platform (Google Forms), consistent with established approaches in recent leishmaniasis KAP research (7, 11). The achieved sample size exceeded commonly recommended thresholds for cross-sectional KAP studies and was considered adequate to detect moderate effect sizes in subgroup analyses. A minimum sample size was estimated using standard cross-sectional formulas, assuming a 50% expected proportion, 95% confidence level, and 5% margin of error, yielding a target sample of approximately 384 participants. The final sample exceeded this threshold.

Potential sources of bias, including selection bias and response bias, were considered during study design and interpretation.

### Data collection tools

Data were collected using a structured, self-administered questionnaire distributed primarily through Google Forms, an online platform consistent with established approaches in recent leishmaniasis KAP research (7, 11). The online format enabled wide geographic coverage across multiple universities simultaneously.

### Sample size calculation

A minimum sample size was estimated using the standard cross-sectional formula: *n* = Z^2^·P·(1-P)/d^2^, where *Z* = 1.96 (95% confidence level), *p* = 0.50 (expected proportion, maximizing required sample), and *d* = 0.05 (margin of error), yielding a minimum target of 384 participants. The achieved sample of 408 exceeded this threshold, providing adequate power to detect moderate effect sizes in subgroup analyses.

### Participants

Participants were recruited using convenience sampling through online distribution channels, including official university WhatsApp and social media academic groups. Probability sampling (e.g., stratified random sampling) was not feasible due to the absence of a comprehensive, accessible sampling frame encompassing all Palestinian medical students across institutions, the decentralized nature of university enrollment databases, and the constraints imposed by the online-only data collection method. The total eligible population could not be precisely determined. Although this proportion is less than a majority of the eligible population, the achieved sample exceeded the calculated minimum sample size of 384 (see Sample Size Calculation), ensuring statistical adequacy for the primary analytical objectives. Convenience sampling through broad online distribution across multiple university networks did facilitate multi-institutional coverage across geographically dispersed universities and diverse training contexts, which would not have been practically achievable through in-person probability sampling given resource and access constraints. This approach introduces acknowledged selection bias, discussed in the Limitations section.

Eligible participants were medical students aged ≥18 years currently enrolled in Palestinian universities. The age threshold of ≥18 years was selected to ensure participants could provide independent informed consent. Year of study was deliberately not used as an eligibility criterion. This decision was intentional: assessing KAP across all training stages, including pre-clinical students, was a primary objective of the study, as inter-stage comparisons were designed to reveal how knowledge and practice evolve across the medical curriculum. Restricting eligibility to clinical-year students would have precluded assessment of the baseline KAP profile and the contribution of early medical education to these outcomes. Pre-clinical students' responses are clearly identified and analyzed separately throughout the Results section. Participants who met the inclusion criteria were excluded from the final analytic dataset if they: (a) did not complete the consent affirmation prior to accessing the questionnaire; (b) submitted responses with >20% missing data across core KAP items; or (c) provided responses with implausible patterns indicative of non-genuine completion (e.g., straight-lining across all items). Non-medical students, visiting/exchange students not enrolled in Palestinian universities, and individuals who declined consent were ineligible and thus not considered part of the target population. Cronbach's alpha values for each domain are reported in the Results section.

### Questionnaire development and translation

The questionnaire was adapted primarily from the validated KAP instrument developed and used by Khan et al. (7), which addresses leishmaniasis and One Health constructs among medical and veterinary professionals, supplemented by items from Qolami-Dastjerdan et al. (11) and Nasiri et al. (12).

The development process followed a content-driven approach similar to published web-based KAP studies, including clear item structures and standardized response options (e.g., Yes/No/Not sure; multiple-choice; multi-response items).

Because the current study required local contextualization, additional items were appended to the original tool to capture clinical decision-making and professional responsibility domains relevant to medical training (e.g., reporting suspected cases, PPE use, and referral/confirmation behaviors).

To ensure linguistic and cultural equivalence, the survey underwent a forward–back translation process with reconciliation of discrepancies, followed by pilot-based refinement (clarity, timing, and acceptability).

Content validity was assessed through expert review by clinicians and researchers with experience in infectious diseases and medical education, who evaluated item relevance and clarity. Construct validity was explored through domain structuring aligned with established KAP frameworks. Internal consistency was evaluated using Cronbach's alpha for each domain, with values ≥0.70 considered acceptable for reliability.

### Scoring and reliability

In contrast, only 12.0% of participants were classified as having good attitude/professional responsibility scores, and only 9.1% had good practice scores, while 51.5% were classified as having poor practice scores. In adjusted models, knowledge was not independently associated with attitude score (B per 10-percentage-point increase = 0.04; 95% CI: −0.03 to 0.11; *p* = 0.227). Scoring followed a priori rules defined in the study scoring guide. Knowledge was initially assessed using core knowledge items coded as correct = 1 and incorrect/“don't know” = 0. After item-level reliability assessment, one item (“most common type in Palestine”) was removed, yielding a final 9-item knowledge score. Attitude/professional responsibility was scored using three items: reporting suspected cases, PPE use when examining skin ulcers, and initial response to a chronic ulcer. Reporting and PPE items were scored as Always = 3, Sometimes = 2, and Never/Don't know = 1; initial clinical response was scored as examine and order smear/biopsy = 3, refer to dermatologist/senior clinician = 2, and antibiotics/traditional remedy/other = 1. The final attitude score ranged from 3 to 9. Practice/environment scores were calculated from four items: evening/night outdoor exposure in sandfly areas, domestic animal proximity, community sandfly-control measures, and participation in a leishmaniasis diagnosis/management course. Higher scores reflected more favorable practice/environmental preparedness. Outdoor exposure was scored as No/Never = 3, Rarely = 2, and Sometimes/Often/Yes = 1; no domestic animal proximity was scored as 3 and yes as 1; community control measures and short-course participation were scored as Yes = 3, Maybe = 2, and No = 1. The final practice score ranged from 4 to 12. For comparability across KAP domains, all composite scores were converted to percentages of the maximum possible domain score and categorized using the same predefined thresholds: poor (< 60%), moderate (60%−79%), and good (≥80%). Accordingly, knowledge was categorized as poor = 0–5, moderate = 6–7, and good = 8–9; attitude as poor = 3–5, moderate = 6–7, and good = 8–9; and practice as poor = 4–7, moderate = 8–9, and good = 10–12.or attitude independently predicted practice score.

### Data management and statistical analysis

Responses were exported from Google Forms into spreadsheet format and subsequently imported into R 4.5.2 (8) for data processing and statistical analysis. Data were cleaned through systematic screening for missing entries, inconsistent coding, and implausible values; where applicable, incomplete or invalid records were excluded from relevant analyses. Categorical variables were summarized as frequencies and percentages, whereas continuous variables were summarized using mean ± standard deviation for approximately normally distributed data, or median and interquartile range for non-normally distributed distributions. Bivariate associations between KAP outcomes and participant characteristics (e.g., sex, residence, university, study stage, and prior training) were assessed using χ^2^ tests (or Fisher's exact test when cell counts were small) for categorical variables, and independent-samples *t*-tests or one-way ANOVA for continuous outcomes; non-parametric alternatives (Mann–Whitney U or Kruskal–Wallis tests) were applied when distributional assumptions were not met. All tests were two-sided with a significance threshold of α = 0.05.

No imputation techniques were applied, as the proportion of missing data was minimal and unlikely to affect the validity of the results.

Multivariable regression models were constructed to identify independent correlates of KAP outcomes and to directly test the KAP framework. Linear regression was used for continuous attitude and practice scores, and logistic regression was used for dichotomized “good” attitude and “good” practice outcomes. First, adjusted models examined the association between knowledge and attitude. Second, practice models included both knowledge and attitude simultaneously to assess their joint influence on practice. Models were adjusted for sex, age, study stage, completion of a parasitology/infectious diseases course, endemic-area origin/residence, and prior awareness of leishmaniasis. Knowledge was entered per 10-percentage-point increase. Adjusted coefficients or adjusted odds ratios were reported with 95% confidence intervals.

This study was reported in accordance with the Strengthening the Reporting of Observational Studies in Epidemiology (STROBE) guidelines, (9) and the completed checklist is provided in the Supplementary material.

### Ethical approval

The study protocol, consent procedure, and survey instrument were reviewed and approved by the Al-Quds University Research Ethics Committee. Participation was voluntary, anonymous, and based on electronic informed consent obtained before questionnaire access; data were de- identified and stored securely with access restricted to the research team.

## Results

Knowledge scores were relatively high, whereas reporting and public health practice indicators were substantially lower.

### Participant characteristics

Due to the online distribution of the survey through open academic networks and social media platforms, the total number of individuals who received the invitation could not be determined; therefore, a response rate could not be calculated.

A total of 408 eligible responses were included in the analytic sample (Table 1). The mean age was 22.18 ± 1.67 years. Participants were predominantly female (64.2%), with 35.5% male and 0.2% missing sex classification. Most respondents resided in cities (52.0%) or villages (27.5%), with smaller proportions from towns (13.2%) and refugee camps (7.4%). In terms of training level, 52.5% were in clinical years (Y4–Y6), 27.7% were pre-clinical (Y1–Y3), and 19.9% were in internship.

**Table 1:** Participant characteristics and epidemiologically relevant exposures (*N* = 408).

Characteristic	Category	*n* (%) or value
A. Demographic characteristics		
Age (years)	Mean ± SD	22.18 ± 1.67
Sex	Female	262 (64.2%)
	Male	145 (35.5%)
	Missing	1 (0.2%)
Residence type	City	212 (52.0%)
	Town	54 (13.2%)
	Village	112 (27.5%)
	Refugee camp	30 (7.4%)
B. Educational characteristics		
Study stage	Pre-clinical (Y1–Y3)	113 (27.7%)
	Clinical (Y4–Y6)	214 (52.5%)
	Internship	81 (19.9%)
University	Al-Quds University	143 (35.0%)
	Palestine Polytechnic University	80 (19.6%)
	An-Najah National University	64 (15.7%)
	Arab American University	45 (11.0%)
	Hebron University	33 (8.1%)
	Universities Abroad (Egypt)	32 (7.8%)
	Islamic University of Gaza	9 (2.2%)
	Universities Abroad (Other)	2 (0.5%)
Completed parasitology/ID course	Yes	311 (76.2%)
	No	97 (23.8%)
First medical student in nuclear family	Yes	212 (52.0%)
	No	196 (48.0%)
Participated in short course on diagnosis/management	Yes	80 (19.6%)
	No	277 (67.9%)
	Maybe	51 (12.5%)
C. Epidemiological exposure and awareness		
Heard about leishmaniasis	Yes	384 (94.1%)
	No	17 (4.2%)
	Unsure	7 (1.7%)
Lives in/Originates from endemic area	Yes	99 (24.3%)
	No	258 (63.2%)
	Maybe	51 (12.5%)
Evening/Night outdoor exposure in sandfly areas	No	81 (19.9%)
	Never	111 (27.2%)
	Rarely	84 (20.6%)
	Sometimes	60 (14.7%)
	Often	18 (4.4%)
	Yes	54 (13.2%)
Domestic animals present near/Inside home	No	211 (51.7%)
	Yes	197 (48.3%)
Community sandfly control measures (Self-reported)	Yes	53 (13.0%)
	No	177 (43.4%)
	Maybe	178 (43.6%)

ID, infectious diseases; SD, standard deviation.

Percentages may not sum to 100% due to rounding. Evening/night outdoor exposure in sandfly areas: “Low/None” combines No + Never + Rarely; “Moderate/High” combines Sometimes + Often + Yes.

Across universities, the largest contributions came from Al-Quds University (35.0%), Palestine Polytechnic University (19.6%), and An-Najah National University (15.7%), with additional representation from other Palestinian institutions and a subset studying abroad (Table 1). Most participants reported having completed a parasitology/infectious diseases course (76.2%). Nearly all respondents had heard about leishmaniasis (94.1%), while 5.9% reported not having heard of it or being unsure. Regarding epidemiological context exposure, 24.3% reported living in/originating from an endemic area, 63.2% reported non-endemic origin/residence, and 12.5% were unsure (“Maybe”). Evening/night outdoor exposure in sandfly areas was commonly reported, with Low/none exposure (No/Never/Rarely) predominating but a substantial subgroup reporting Moderate/high exposure (Sometimes/Often/Yes) (Table 1).

### Knowledge scale internal consistency and distribution

In accordance with the scoring framework, the core knowledge scale was evaluated for internal consistency, and one item was removed to achieve the target reliability threshold (α ≥ 0.74) as specified by the scoring system. The resulting 9-item knowledge scale demonstrated acceptable internal consistency (Cronbach's α = 0.747). Cronbach's alpha was reported for the knowledge domain because the knowledge items were designed to function as a unified reflective scale assessing factual understanding of leishmaniasis. In contrast, the attitude and practice domains included conceptually heterogeneous items reflecting distinct professional responsibilities and behaviors, such as reporting suspected cases, use of personal protective measures, referral or diagnostic decision-making, and environmental exposure-related practices. These items were therefore treated as descriptive indicators rather than as a single internally consistent latent scale. Because Cronbach's alpha assumes that items measure the same underlying construct, calculating alpha for these multidimensional domains could be misleading. This clarification has now been added to the Methods section.

Across the sample, the mean knowledge score was 6.57 ± 2.20 (out of 9), corresponding to 72.98% (95% CI: 70.60%−75.37%). The median was 7 (IQR: 5–8). Using percentage-based cutoffs aligned with the score categorization thresholds defined in the scoring framework, 44.4% (*n* = 181) met the “Good” knowledge threshold, 27.5% (*n* = 112) were “Moderate,” and 28.2% (*n* = 115) were classified as “Poor.”

### Attitude and practice indicators

Across professional responsibility indicators, glove/PPE use during examination of skin ulcers was high: Always in 65.0% (*n* = 265), Sometimes in 23.8% (*n* = 97), and Never in 11.3% (*n* = 46). In contrast, reporting suspected cases was much less frequent: Always in 7.6% (*n* = 31), Sometimes in 15.4% (*n* = 63), and Never in 52.5% (*n* = 214); additionally, 24.5% (*n* = 100) reported not knowing the reporting system.

Regarding first clinical response to a chronic ulcer, 51.7% (*n* = 211) selected “examine and order smear/biopsy,” and 37.5% (*n* = 153) selected referral to a dermatologist/senior clinician; smaller proportions selected antibiotics/topicals (4.9%), traditional remedies (2.2%), or other actions (3.7%).

Subgroup analyses of attitude scores (professional responsibility composite) revealed no statistically significant variation across sex, study stage, or university affiliation (Table 2), suggesting that professional responsibility attitudes toward leishmaniasis are uniformly distributed regardless of training level. This is a notable finding, as it implies that even clinical-stage students, despite higher knowledge, do not demonstrate correspondingly higher professional responsibility attitudes, further evidencing the knowledge–attitude disconnect. Practice/environment scores, however, differed significantly by outdoor exposure level (*p* < 0.001) and domestic animal proximity (*p* < 0.001), consistent with the hypothesis that environmental exposure shapes preventive practice behaviors independently of formal knowledge acquisition.

**Table 2:** Knowledge, attitude, and practice scores across demographic, educational, and exposure variables.

Characteristic/Group		Knowledge score (%)		Attitude score (3–9)		Practice score (4–12)	
		Mean ±SD	*p-value*	Mean ±SD	*p-value*	Mean ±SD	*p-value*
A. Demographic characteristics							
Sex	Female	74.17 ± 24.31	0.177	6.29 ± 1.25	0.441	7.37 ± 1.70	0.844
	Male	70.73 ± 24.77	—	6.19 ± 1.33	—	7.41 ± 1.55	—
Age group	19–21 years	60.23 ± 27.05	< 0.001	6.42 ± 1.20	0.146	7.60 ± 1.78	0.188
	22–23 years	78.37 ± 21.62	—	6.14 ± 1.30	—	7.24 ± 1.60	—
	24–25 years	80.37 ± 18.67	—	6.21 ± 1.32	—	7.38 ± 1.57	—
Residence type	City	74.63 ± 23.11	0.278	6.26 ± 1.25	0.478	7.49 ± 1.73	0.494
	Town	74.28 ± 24.78	—	6.04 ± 1.45	—	7.31 ± 1.43	—
	Village	71.43 ± 25.34	—	6.28 ± 1.29	—	7.21 ± 1.60	—
	Refugee camp	64.81 ± 29.05	—	6.47 ± 1.01	—	7.57 ± 1.77	—
B. Educational characteristics							
Study stage	Pre-clinical (Y1–Y3)	55.95 ± 26.68	< 0.001	6.30 ± 1.24	0.864	7.67 ± 1.85	0.139
	Clinical (Y4–Y6)	79.60 ± 20.02	—	6.25 ± 1.24	—	7.30 ± 1.63	—
	Internship	79.29 ± 20.47	—	6.20 ± 1.43	—	7.25 ± 1.39	—
University	Al-Quds University	71.17 ± 25.75	0.708	6.32 ± 1.25	0.678	7.55 ± 1.86	0.251
	Palestine Polytechnic Univ.	75.42 ± 22.41	—	6.05 ± 1.12	—	7.18 ± 1.49	—
	An-Najah National Univ.	72.74 ± 26.18	—	6.25 ± 1.31	—	7.73 ± 1.83	—
	Arab American Univ.	70.86 ± 25.10	—	6.16 ± 1.37	—	7.16 ± 1.50	—
	Hebron University	70.71 ± 22.54	—	6.18 ± 1.19	—	7.24 ± 1.76	—
	Islamic Univ. of Gaza	79.01 ± 17.07	—	6.56 ± 1.67	—	7.67 ± 1.41	—
	Universities Abroad (Egypt)	79.17 ± 23.91	—	6.09 ± 1.44	—	7.00 ± 1.44	—
	Universities Abroad (Other)	72.22 ± 7.86	—	5.50 ± 0.71	—	7.50 ± 0.71	—
Completed parasitology/ID course	Yes	79.81 ± 19.66	< 0.001	6.24 ± 1.28	0.749	7.37 ± 1.67	0.584
	No	51.09 ± 25.64	—	6.29 ± 1.27	—	7.47 ± 1.63	—
First medical student in family	Yes	73.76 ± 23.62	0.422	6.28 ± 1.24	0.601	7.39 ± 1.66	0.950
	No	72.14 ± 25.37	—	6.23 ± 1.31	—	7.40 ± 1.66	—
Short course on diagnosis/management	Yes	70.28 ± 24.48	**0.028**	6.40 ± 1.35	0.093	8.86 ± 1.58	< 0.001
	No	75.45 ± 23.28	—	6.11 ± 1.22	—	7.05 ± 1.53	—
	Maybe	63.83 ± 28.44	—	6.33 ± 1.29	—	7.29 ± 1.58	—
C. Epidemiological exposure							
Lives in/Originates from endemic area	Yes	83.05 ± 17.05	< 0.001	6.38 ± 1.16	0.371	7.40 ± 1.59	0.889
	No	71.96 ± 24.47	—	6.21 ± 1.29	—	7.41 ± 1.72	—
	Maybe	58.61 ± 28.55	—	6.33 ± 1.29	—	7.31 ± 1.58	—
Evening/Night outdoor exposure (Sandfly areas)	Low/None	74.03 ± 22.95	0.092	6.29 ± 1.23	0.337	7.77 ± 1.56	< 0.001
	Moderate/High	70.80 ± 27.35	—	6.18 ± 1.36	—	6.61 ± 1.60	—
Domestic animals near/Inside home	Yes	71.52 ± 25.31	0.265	6.33 ± 1.29	0.209	6.08 ± 1.34	< 0.001
	No	74.36 ± 23.71	—	6.18 ± 1.26	—	8.62 ± 1.24	—
Community sandfly control measures	Yes	72.72 ± 23.72	0.929	6.34 ± 1.29	0.718	9.10 ± 1.16	< 0.001
	No	72.60 ± 25.01	—	6.27 ± 1.25	—	6.93 ± 1.60	—
	Maybe	73.46 ± 24.24	—	6.21 ± 1.29	—	7.53 ± 1.52	—

All values are presented as Mean ± SD. Bold p-values indicate statistical significance at α = 0.05. For variables with three or more groups, a single omnibus p-value (Welch's ANOVA or Kruskal–Wallis) is shown on the first group row; subsequent group rows are marked “—.” For two-group variables, an independent-samples t-test or Mann–Whitney U p-value is shown on the first group row. Knowledge score (%): 0–100 continuous percentage; categorized as Poor (< 60%), Moderate (60%−79%), Good (≥80%). Attitude score: professional responsibility / attitude composite (range 3–9). Practice score: practice/environment composite (range 4–12).

ID, infectious diseases; Univ., University; SD, standard deviation.

Overall, the professional responsibility/attitude composite score averaged 6.25 ± 1.27 out of 9 (95% CI: 6.13–6.38). Using the predefined thresholds, 12.0% (*n* = 49) were classified as having good attitude/professional responsibility scores, 68.1% (*n* = 278) as moderate, and 19.9% (*n* = 81) as poor.

The practice/environment composite averaged 7.39 ± 1.66 out of 12 (95% CI: 7.23–7.56). Only 9.1% (*n* = 37) were classified as having good practice scores, 39.5% (*n* = 161) as moderate, and 51.5% (*n* = 210) as poor. Thus, although 44.4% of participants achieved good knowledge scores, substantially fewer achieved good attitude or practice scores, highlighting a clear gap between knowledge and applied professional/public health behaviors.

### Knowledge differences by training stage and age

Knowledge levels varied strongly by stage of training (Table 2). The mean knowledge percentage was 55.95% ± 26.68 in pre-clinical students, compared with 79.60% ± 20.02 in clinical students and 79.29% ± 20.47 in interns. The overall difference across stages was statistically significant [Welch's *F*_(2, 185.2)_ = 36.05, *p* < 0.001], with a large proportion of explained variance (η^2^ = 0.186). Pairwise comparisons showed that pre-clinical students scored substantially lower than clinical students (mean difference = 23.65 percentage points, 95% CI: 18.01–29.29; Hedges' *g* = 1.05; Bonferroni-adjusted *p* < 0.001) and interns (mean difference = 23.34 points, 95% CI: 16.66–30.02; *g* = 0.96; adjusted *p* < 0.001); there was no meaningful difference between clinical students and interns (*p* = 0.908).

Similarly, knowledge differed significantly across age groups (Table 2). Participants aged 19–21 averaged 60.23% ± 27.05, while those aged 22–23 averaged 78.37% ± 21.62 and those aged 24–25 averaged 80.37% ± 18.67 [Welch's *F*_(2, 253.8)_ = 26.20, *p* < 0.001; η^2^ = 0.133]. Pairwise comparisons indicated that both older groups scored higher than the 19–21 group (both Bonferroni-adjusted *p* < 0.001), while the 22–23 and 24–25 groups did not differ materially (*p* = 0.416). This represents a substantial and practically meaningful difference, highlighting the strong influence of clinical exposure on knowledge acquisition.

Values are mean ± SD. *P-values* correspond to between-group comparisons within each characteristic.

### Knowledge differences by coursework completion and epidemiological exposure

Completion of a parasitology/infectious diseases course was associated with markedly higher knowledge: 79.81% ± 19.66 among completers vs. 51.09% ± 25.64 among non-completers [Welch's *t*_(133.0)_ = 10.14, *p* < 0.001], with a large effect size (Hedges' *g* = 1.35) and a mean difference of 28.73 percentage points (95% CI: 23.12–34.33).

Knowledge also differed by self-reported endemic-area origin/residence. Those reporting endemic exposure (“Yes”) averaged 83.05% ± 17.05, compared with 71.96% ± 24.47 among “No” and 58.61% ± 28.55 among “Maybe” [Welch's *F*_(2, 123.5)_ = 21.22, *p* < 0.001; η^2^ = 0.085].

The “Yes” group exceeded the “No” group (mean difference = 11.09 points, 95% CI: 6.57–15.60; *p* < 0.001) and the “Maybe” group (difference = 24.45 points, 95% CI: 15.77–33.12; *p* < 0.001).

Participants who reported having heard about leishmaniasis scored higher than those who had not/unsure (74.91% ± 23.38 vs. 42.13% ± 21.23; Welch's *t*_(26.6)_ = 7.29, *p* < 0.001; *g* = 1.41; mean difference = 32.78 points, 95% CI: 23.56–42.01). This contrast should be interpreted with caution due to the small size of the “No/Unsure” subgroup (*n* = 24) but remained highly statistically significant.

### Comparisons by sex, residence, and university

No statistically significant knowledge differences were detected by sex (*p* = 0.177) or residence type (*p* = 0.278, Table 2). Knowledge also did not differ significantly across university categories (Welch *p* = 0.708), despite variation in institution-specific means (range approximately 70.7%−79.2%). In parallel, neither attitude nor practice scores showed consistent statistically significant variation across sex, residence, training stage, or university (Table 2), with most *p-values* exceeding 0.05.

### Distributional differences in knowledge categories

Knowledge category distributions differed strongly by training stage and course completion. The proportion classified as “Poor” knowledge was highest among pre-clinical students (60.2%) and lowest among clinical students (14.0%) (χ^2^ association detailed in Table 2 comparisons and supported by category counts). For course completion, “Poor” knowledge comprised 73.2% among non-completers vs. 14.1% among completers, indicating a pronounced distributional shift.

### Item-level performance on core and clinical knowledge indicators

Within the 9-item core knowledge set, the highest correct response rates were observed for infectiousness of leishmaniasis (92.4%) and treatability (91.4%). Correct understanding of vector identity (sandfly) was also high (75.2%), as was correct identification of a confirmatory diagnostic approach (skin smear/biopsy; 73.3%) and the causative agent (protozoa; 71.6%). In contrast, knowledge of reservoirs (dogs and rodents) was comparatively low (46.8% correct), representing the weakest core item (Table 3).

**Table 3:** Item-level performance and stage-based comparisons for core knowledge and selected clinical indicators.

Knowledge item	Overall % correct	Pre-clinical %	Clinical %	Internship %	*χ^2^ (df)*	*p*
A. Core knowledge items (9-item scale)						
Leishmaniasis is an infectious disease	92.4	91.2	92.1	95.1	1.10 (2)	0.576
Causative agent is a protozoan parasite	71.6	39.8	84.6	81.5	77.68 (2)	< 0.001
Transmission via sandfly bite	68.4	74.3	69.2	58.0	5.93 (2)	0.052
Leishmaniasis is a zoonotic disease	68.1	43.4	76.6	80.2	44.54 (2)	< 0.001
Vector is the sandfly (phlebotomine)	75.2	56.6	84.6	76.5	31.09 (2)	< 0.001
Reservoirs include dogs and rodents	46.8	24.8	56.1	52.0	27.23 (2)	< 0.001
Visceral form affects liver and spleen	69.6	55.8	75.7	77.8	14.63 (2)	**0.001**
Confirmatory diagnosis: skin smear/biopsy	73.3	61.1	77.1	82.7	11.61 (2)	**0.003**
Leishmaniasis is a treatable disease	91.4	86.7	93.5	95.1	4.24 (2)	0.120
B. Advanced/Clinical knowledge items						
Most reliable confirmation method (microscopy/tissue)	65.0	42.5	75.2	71.6	23.91 (2)	< 0.001
Optimal specimen: skin scraping/biopsy	78.2	64.6	83.6	85.2	14.44 (2)	**0.001**
Clinical diagnosis alone is insufficient (correct = No)	52.5	36.3	59.3	60.5	16.04 (2)	< 0.001
Visceral findings triad: splenomegaly + pancytopenia + amastigotes	23.3	16.8	24.8	29.6	3.50 (2)	0.174
Correct treatment drugs without error (antimonials + amphotericin B + miltefosine only)	16.9	9.7	20.6	18.5	7.24 (2)	**0.027**
All major diagnostic modalities selected (smear + culture + PCR + serology)	18.4	12.4	21.5	16.0	4.87 (2)	0.088

Bold p-values indicate statistical significance at α = 0.05. χ^2^ tests (2 df) were used for all between-group comparisons. Pre-clinical = Years 1–3; Clinical = Years 4–6; Internship = Year 7+.

PCR, polymerase chain reaction.

Assessment of advanced/clinical knowledge items indicated adequate recognition of specimen and microscopy concepts, but low rates of fully correct multi-component answers. Specifically, the “most reliable confirmation method” was answered correctly by 65.0%, and correct identification of the typical specimen “skin scraping/biopsy” was observed in 78.2%. However, only 52.5% correctly rejected “clinical diagnosis alone” as sufficient confirmation. The proportion achieving a fully correct response for the classic visceral leishmaniasis findings triad (splenomegaly + pancytopenia + demonstration of amastigotes) was 23.3%, and only 16.9% provided a fully correct treatment drug selection without incorrect agents; a similarly low proportion (18.4%) selected all major diagnostic test modalities (smear + culture + PCR + serology) without omission.

Training stage differences were substantial for multiple core and advanced items. Pre-clinical students showed markedly lower correct response rates for the causative agent (39.8% vs. 84.6% clinical) and zoonosis concept (43.4% vs. 76.6% clinical), both with large χ^2^ associations (Table 3). For several items, clinical and internship groups were comparable, while pre-clinical performance lagged.

### Self-assessed knowledge vs. measured knowledge

Self-rated knowledge (1–5) had no meaningful association with measured knowledge. Pearson correlation between self-rated and actual knowledge percentage was *r* = −0.047 (*p* = 0.341), and Spearman correlation was ρ = −0.043 (*p* = 0.384).

### Correlations among KAP domains

Knowledge percentage showed only a weak positive correlation with the attitude/professional responsibility composite (*r* = 0.073) and a weak negative correlation with the practice/environment composite (*r* = −0.055). Attitude and practice scores were essentially uncorrelated (*r* = −0.011). These weak unadjusted associations were further evaluated using adjusted regression models.

### Multivariable predictors of knowledge and good knowledge classification

In multivariable linear regression (Table 4), several predictors remained independently associated with higher knowledge percentage. After adjustment, completing a parasitology/infectious diseases course was associated with a +19.55 percentage point increase in knowledge (95% CI: 12.62–26.49; *p* < 0.001). Being in pre-clinical training was associated with lower knowledge relative to clinical stage (*B* = −9.62; 95% CI: −17.83 to −1.40; *p* = 0.022). Endemic-area origin/residence (“Yes” vs. “Maybe”) was independently associated with higher knowledge (*B* = +13.39; 95% CI: 4.68–22.09; *p* = 0.003). Having heard about leishmaniasis (Yes vs. No/Unsure) was also associated with higher knowledge (*B* = +25.55; 95% CI: 13.70–37.40; *p* < 0.001). Sex and age were not significant predictors in the adjusted model.

**Table 4:** Multivariable models for knowledge (Linear) and “Good” knowledge classification (Logistic).

Predictor	Linear regression (Knowledge %)		Logistic regression (“Good” Knowledge)	
	*B* (95% CI)	*p*	aOR (95% CI)	*p*
A. Demographic predictors				
Male (vs. Female)	−2.78 (−6.76, 1.19)	0.170	0.88 (0.56, 1.39)	0.586
Age (per 1-year increase)	0.69 (−1.91, 3.28)	0.605	0.95 (0.73, 1.24)	0.724
B. Training stage (reference = Clinical)				
Pre-clinical (vs. Clinical)	−9.62 (−17.83, −1.40)	**0.022**	0.53 (0.23, 1.21)	0.130
Internship (vs. Clinical)	−0.58 (−7.36, 6.20)	0.866	1.13 (0.57, 2.27)	0.723
C. Educational and epidemiological predictors				
Completed parasitology/ID course (Yes vs. No)	+19.55 (12.62, 26.49)	< 0.001	3.37 (1.70, 6.67)	< 0.001
Non-endemic origin/residence (No vs. Maybe)	7.49 (−0.93, 15.92)	0.081	2.13 (0.97, 4.65)	0.059
Endemic origin/residence (Yes vs. Maybe)	+13.39 (4.68, 22.09)	**0.003**	3.43 (1.48, 7.95)	**0.004**
Heard about leishmaniasis (Yes vs. No/Unsure)	+25.55 (13.70, 37.40)	< 0.001	16.27 (2.10, 126.25)	**0.008**

Bold p-values indicate statistical significance at α = 0.05. B, unstandardised regression coefficient (percentage points); aOR, adjusted odds ratio; CI, confidence interval; ID, infectious diseases. Linear model outcome: continuous knowledge percentage. Logistic model outcome: Good knowledge (≥80%) vs. Poor/Moderate (< 80%). Both models adjusted for all predictors listed. Reference categories: Female (sex); Clinical stage (training stage); Maybe (endemic area).

In adjusted logistic regression (Table 4), completing the parasitology course increased the odds of being classified as “Good knowledge” by approximately 3.37-fold (aOR = 3.37; 95% CI: 1.70–6.67; *p* < 0.001). Endemic-area origin/residence (“Yes” vs. “Maybe”) was also associated with higher odds (aOR = 3.43; 95% CI: 1.48–7.95; *p* = 0.004). Reporting having heard about leishmaniasis showed a large odds ratio (aOR = 16.27; 95% CI: 2.10–126.25; *p* = 0.008), reflecting strong separation between groups but also wide uncertainty consistent with the small size of the No/Unsure subgroup. Study stage did not reach statistical significance in the adjusted logistic model, although the direction suggested lower odds among pre-clinical respondents.

Among all predictors, completion of a parasitology/infectious diseases course demonstrated the strongest and most consistent association with higher knowledge levels.

Adjusted analyses examining the association between knowledge and practice/attitude scores demonstrated weak correlations, reinforcing the presence of a knowledge–practice gap.

### Adjusted KAP pathway models

In the adjusted linear model with attitude score as the outcome (*n* = 407), knowledge was not independently associated with attitude/professional responsibility score (B per 10-percentage-point increase = 0.04; 95% CI: −0.03 to 0.11; *p* = 0.227). A logistic model using good attitude classification produced a similar finding (aOR per 10-percentage-point increase in knowledge = 1.02; 95% CI: 0.87–1.20; *p* = 0.798).

In the practice model including both knowledge and attitude, neither domain independently predicted practice/environment score after adjustment. Knowledge showed no significant association with practice score (B per 10-percentage-point increase = −0.04; 95% CI: −0.12 to 0.05; *p* = 0.391), and attitude score was also not associated with practice score (B per 1-point increase = −0.01; 95% CI: −0.14 to 0.12; *p* = 0.884). Logistic regression for good practice classification showed the same pattern: knowledge (aOR = 0.91; 95% CI: 0.75–1.11; *p* = 0.339) and attitude score (aOR = 1.15; 95% CI: 0.86–1.55; *p* = 0.349) were not significant predictors. These findings indicate that higher knowledge and more favorable professional responsibility attitudes did not reliably translate into better practice scores (Table 5).

**Table 5A:** Categorized KAP domains and adjusted KAP pathway models.

Domain	Score range	Poor (%)	Moderate (%)	Good (%)
Part A. Categorized KAP domain scores				
**Categorized KAP domain scores (*****N*** = **408)**				
Knowledge	0–9 (items)	115 (28.2%)	112 (27.5%)	181 (44.4%)
Attitude/Professional responsibility	3–9 (points)	81 (19.9%)	278 (68.1%)	49 (12.0%)
Practice/Environment	4–12 (points)	210 (51.5%)	161 (39.5%)	37 (9.1%)

**Table 5B:** 

Outcome	Predictor	Effect (B or aOR)	95% CI	*p-value*
Part B. Adjusted KAP pathway models				
**A. Knowledge as predictor of attitude (Adjusted linear and logistic models)**				
Attitude score (continuous)	Knowledge, per 10 percentage-point increase	*B* = 0.04	−0.03 to 0.11	0.227
Good attitude (binary)	Knowledge, per 10 percentage-point increase	aOR = 1.02	0.87 to 1.20	0.798
**B. Knowledge and attitude as joint predictors of practice (Adjusted Models)**				
Practice score (continuous)	Knowledge, per 10 percentage-point increase	*B* = −0.04	−0.12 to 0.05	0.391
	Attitude score, per 1-point increase	*B* = −0.01	−0.14 to 0.12	0.884
Good practice (binary)	Knowledge, per 10 percentage-point increase	aOR = 0.91	0.75 to 1.11	0.339
	Attitude score, per 1-point increase	aOR = 1.15	0.86 to 1.55	0.349

Categories were defined as poor < 60%, moderate 60%−79%, and good ≥80% of the maximum possible domain score. Adjusted models controlled for sex, age, study stage, completion of parasitology/infectious diseases coursework, endemic-area origin/residence, and prior awareness of leishmaniasis. Adjusted model n = 407 because one participant had missing sex.

## Discussion

Leishmaniasis remains a significant neglected tropical disease affecting multiple regions worldwide, including the Middle East and parts of Palestine. Assessing the level of awareness among future healthcare providers is essential for improving early diagnosis, prevention, and disease control strategies (9). This cross-sectional study was conducted to evaluate the knowledge, attitudes, and practices (KAP) related to leishmaniasis among medical students in Palestine.

Consistent with findings reported by Qolami-Dastjerdan et al. (11) and Nasiri et al. (2020) (12), the present study demonstrated an overall satisfactory level of knowledge (72.98%). However, significant variability was observed across subgroups, primarily associated with level of training and completion of parasitology-related coursework. Notably, a clear gap between knowledge and actual practices was identified.

These findings are consistent with previous KAP studies conducted in endemic regions such as Iran and Morocco, which have similarly reported moderate to high knowledge levels accompanied by deficiencies in preventive practices and reporting behaviors (10–12, 14, 15). This pattern suggests that the gap between knowledge acquisition and practical application may represent a broader systemic issue in medical education within endemic settings rather than a context-specific phenomenon.

Knowledge levels were significantly influenced by the stage of clinical training, with clinical students and interns demonstrating higher scores (79.60% and 79.29%, respectively) compared to pre-clinical students (55.95%). These findings are consistent with previous studies conducted in Iran (12). Furthermore, students who had completed parasitology or infectious disease courses achieved substantially higher knowledge scores (79.81%) compared to those who had not (51.09%). Together, these findings emphasize the importance of structured academic exposure and the early integration of parasitology content within medical curricula to bridge knowledge gaps related to tropical and vector-borne diseases (11, 12).

Exposure to endemic areas was also associated with higher baseline understanding. Participants who reported living in or originating from endemic regions demonstrated higher scores (83.05%) compared to those without such exposure (71.96%) or those uncertain about their exposure (58.61%). This likely reflects the role of environmental exposure and personal experience in shaping disease awareness (13). Similarly, prior awareness of leishmaniasis was associated with significantly higher knowledge levels (74.91% vs. 42.13%), suggesting that baseline familiarity contributes to improved understanding (10). Age-related differences were also observed, with participants aged 24–25 demonstrating the highest knowledge levels (80.37%), compared to younger groups. In contrast, no significant differences were identified based on sex, residence, or university affiliation, partially aligning with previous findings, although some studies have reported sex-based differences (10).

Regarding specific knowledge indicators, a large proportion of participants correctly identified key aspects of the disease, including its infectious nature (92.4%), vector (75.2%), and transmission through sandfly bites (68.4%). These findings are comparable to prior studies conducted in Iran and other regions (10, 12, 13). Additionally, 71.6% correctly identified the causative agent as a protozoan parasite, and 68.1% recognized the zoonotic nature of the disease.

Awareness of treatability was high (91.4%), and 73.3% correctly identified skin smear or biopsy as a confirmatory diagnostic method. These results are consistent with regional KAP studies, including those conducted in Pakistan and Morocco (7, 14).

Despite generally acceptable knowledge levels, important gaps were identified in more advanced clinical domains. Only 16.9% of participants correctly identified appropriate treatment options without error, and just 18.4% selected all major diagnostic modalities (smear, culture, PCR, and serology). Knowledge of infection reservoirs was also limited, with only 46.8% correctly identifying animals such as dogs and rodents. Similar findings have been reported by Bouyahya et al. (15), highlighting deficiencies in understanding transmission cycles, reservoirs, and preventive strategies.

In terms of practices, a clear discrepancy between knowledge and behavior was observed. Although adherence to personal protective measures, such as glove use, was relatively high (65.0%), reporting of suspected leishmaniasis cases was markedly inadequate, with only 7.6% consistently reporting cases and 24.5% unaware of reporting systems. This gap between individual protective behavior and broader public health responsibilities may reflect insufficient training in disease surveillance systems and limited clinical exposure. Comparable findings have been reported in previous studies, including those by Khan et al. (7), Qolami-Dastjerdan et al. (11), and Bouyahya et al. (15), all of which identified similar gaps between knowledge and applied practices.

With respect to attitudes, the observed uniformity across subgroups is particularly noteworthy. Despite the wide range of knowledge levels, particularly between pre-clinical and clinical students, professional responsibility attitudes did not vary significantly by training stage (*p* = 0.864), suggesting that medical education in its current form does not effectively shape professional responsibility attitudes toward reporting endemic diseases even as knowledge increases. This aligns with the Health Belief Model (17), which identifies perceived responsibility and self-efficacy, rather than knowledge *per se*, as the primary drivers of health-protective behavior. Educational interventions specifically targeting professional norms and reporting obligations may therefore be more effective than knowledge-focused curricula alone.

With respect to clinical decision-making, slightly more than half of participants (51.7%) selected appropriate initial diagnostic actions, such as examination and ordering a smear or biopsy, while 37.5% preferred referral to a specialist. However, a minority reported inappropriate approaches, including empirical antibiotic use or reliance on traditional remedies, indicating the persistence of misconceptions regarding management. Previous case-based studies emphasize the importance of timely and accurate diagnosis in improving patient outcomes in cutaneous leishmaniasis (12).

Although levels of knowledge were relatively high, attitudes were comparatively less optimal. In contrast, Nasiri et al. (12) reported more favorable attitudes among participants. No significant differences in attitudes or practices were observed across demographic variables, suggesting that these gaps may be widespread rather than confined to specific subgroups. This aligns with prior research indicating that knowledge and practice variability is more strongly influenced by targeted training than by demographic characteristics (12, 14).

Overall, participants demonstrated generally appropriate clinical responses and safe individual practices in the context of suspected infectious disease. However, substantial deficiencies were observed in formal reporting practices and integration of public health responsibilities into clinical behavior. Strengthening training in disease notification systems, epidemiological understanding, and applied clinical management through targeted educational interventions is likely to improve both individual patient care and broader public health outcomes.

An important finding of this study was that the KAP domains did not follow a simple linear pathway. Although knowledge scores were relatively high, only 12.0% of participants achieved good attitude/professional responsibility scores and only 9.1% achieved good practice scores. Moreover, adjusted regression models showed that knowledge was not independently associated with attitude, and that knowledge and attitude jointly did not predict practice. This strengthens the evidence for a knowledge–attitude–practice gap: theoretical knowledge alone appears insufficient to shape professional responsibility or applied public health behaviors. These findings are consistent with previous KAP studies reporting persistent gaps between knowledge and applied practices (7, 11, 15), and also align with the Knowledge–Behavior Gap (16) and the Health Belief Model (17), which emphasize that knowledge does not necessarily translate into action without perceived responsibility, self-efficacy, system awareness, and practical reinforcement. Educational interventions should therefore go beyond knowledge delivery and include practical reporting exercises, simulation-based case management, explicit training in notification systems, and reinforcement of professional responsibility toward endemic disease surveillance.

Furthermore, although 94.1% of participants reported prior awareness of leishmaniasis, gaps persisted in more complex clinical domains, particularly regarding treatment options and the diagnosis of visceral leishmaniasis in adults. Similar discrepancies have been reported in previous KAP studies among medical and health sciences students, where high general awareness does not necessarily translate into detailed clinical competence or optimal practices (18).

The observed disconnect between knowledge and practice highlights a critical limitation of traditional knowledge-based medical education models (16). While theoretical instruction may improve cognitive understanding, it does not necessarily translate into behavioral competence, particularly in areas requiring system-level awareness such as disease surveillance and reporting. This finding underscores the importance of integrating experiential learning, simulation-based training, and public health system exposure into undergraduate medical curricula.

These findings have important implications for medical education and public health policy. Integrating leishmaniasis education into clinical training, incorporating simulation-based learning, and providing structured instruction on disease reporting systems may enhance preparedness. Additionally, strengthening collaboration between medical schools and public health authorities could improve awareness of surveillance protocols and reporting responsibilities among future healthcare providers.

More broadly, this also highlights a systemic challenge in bridging clinical education with public health responsibilities in endemic regions. Addressing this gap may require not only curriculum-level changes but also structural integration of surveillance training, interdisciplinary collaboration, and alignment between academic institutions and national health systems.

Despite relatively high knowledge levels, the failure to translate knowledge into consistent reporting behavior represents a critical vulnerability in the clinical–public health interface of future healthcare systems in endemic regions.

## Limitations

The cross-sectional design of this study limits the ability to establish causal relationships between participant characteristics and KAP outcomes. Additionally, the use of a self-administered questionnaire introduces the potential for information and social desirability bias, as participants may overestimate their knowledge or report idealized behaviors. The use of convenience sampling through online distribution may have also introduced selection bias, potentially overrepresenting more motivated or academically engaged students. Non-response bias may also have influenced the findings. Furthermore, although the questionnaire was adapted from existing tools, external validation in this specific context was limited. Residual confounding may persist despite multivariable adjustment. Although the study included multiple universities, the findings reflect a specific educational and epidemiological context and may not be fully generalizable to other regions. Furthermore, certain potentially relevant factors, such as prior clinical exposure to leishmaniasis cases or participation in public health initiatives, were not assessed and may influence KAP outcomes.

Additionally, as with all KAP studies, responses may not fully reflect actual behavior in real clinical settings, limiting the ability to infer real-world practice patterns.

Moreover, the absence of objective measures of clinical competence limits the ability to confirm whether self-reported practices accurately reflect real-world clinical behavior.

## Conclusion

This study demonstrates that medical students in Palestine possess a generally good level of knowledge regarding leishmaniasis, particularly in relation to its basic characteristics, transmission, and diagnosis. Higher knowledge levels were associated with advanced clinical training, completion of parasitology coursework, and exposure to endemic areas, underscoring the importance of both education and experiential learning. However, no significant differences were observed in practice patterns across demographic groups, indicating widespread deficiencies in applied skills and public health engagement.

Overall, these findings highlight that improving knowledge alone is insufficient to ensure effective clinical and public health practice. Bridging the gap between knowledge and action may require a shift toward competency-based medical education that integrates clinical decision-making, surveillance awareness, and real-world application. The categorization of all KAP domains further clarified the gap: good knowledge was substantially more common than good attitude or good practice, and more than half of participants were classified as having poor practice scores. Adjusted KAP pathway models confirmed that knowledge and attitude did not independently translate into better practice, underscoring the need for competency-based and system-oriented training rather than knowledge-focused teaching alone. Further research is needed to evaluate the effectiveness of such interventions in improving both individual patient outcomes and broader public health responses in endemic settings.

## Data Availability

The raw data supporting the conclusions of this article will be made available by the authors, without undue reservation.
